# A Case of Pemphigus

**Published:** 1792

**Authors:** T. M. Winterbottom

**Affiliations:** Physician to the Settlement at Sierra Leone.


II. *A Caſe of Pemphigus. By* T. M. Winterbottom, *M. D. Phyſician to the Settlement at Sierra Leone.*

MR. Y., aged eighteen years, of a ſallow complexion, but healthy, in the ſummer of 1790, made a voyage to Archangel. On his arrival at that place he was, without any previous uneaſineſs, affected with an eruption of ſmall veſicles, of the ſize of peaſe, which gradually increaſed to the ſize of large hazle nuts: they continued in this form for ſome time, then burſt, and diſcharged a thin fluid of a light yellow colour, like an infuſion of green tea, leaving the ſkin beneath excoriated, and in a very ſenſible ſtate. Theſe veſicles affected in particular his face and legs; they did not obſerve any regular period, but as one ſet of them diſappeared others broke out afreſh.

Theſe ſucceſſive eruptions continued during the whole time of his ſtay at Archangel, and he was not entirely free from them until he had paſſed the North Cape on his paſſage home. The only application made uſe of was a little cerate, to prevent the cloths from ſticking to the wounds, and he took a dofe or two of Glauber's falt.

But the peculiarity of this cafe is, that the very fame train of fymptoms took place upon a fecond voyage to Archangel in 1791. The veficles came out in like manner, making their appearance in fucceffion; nor did the eruption ceafe till he had again paffed the North Cape.

When I firft faw him, which was fome months after this fecond voyage, his face and legs were covered with fpots nearly the fize of a fixpence, in colour refembling the marks left by the fmall pox; many of thefe were attended with confiderable depreffions of the fkin, infomuch as to produce a fufpicion, among perfons not much acquainted with the difeafe, of his really having had the fmall pox. The difcolouration of the fkin was not entirely removed for a twelvemonth after the difeafe had left him, but was ftill very evident in cold weather. None of the people in the fhip with him were affected in the fame manner; neither could the complaint be referred to the bites of mufquitoes or other infects, fince he had been at St. Peterfburgh and other places equally infefted with them without experiencing the leaft inconvenience.

